# Immunological discrepancy in aged mice facilitates skin allograft survival

**DOI:** 10.18632/aging.203152

**Published:** 2021-06-22

**Authors:** Wei-Chen Lee, Yu-Chao Wang, Hsiu-Ying Hsu, Pao-Yueh Hsu, Chih-Hsien Cheng, Chen-Fang Lee, Ting-Jung Wu, Kun-Ming Chan

**Affiliations:** 1 Division of Liver and Transplantation Surgery, Department of General Surgery, Chang-Gung Memorial Hospital, Chang-Gung University College of Medicine, Taoyuan, Taiwan

**Keywords:** aged recipient, organ transplantation, immunosuppression, myeloid-derived suppressor cell

## Abstract

More and more aged people are undergoing organ transplantation. Understanding aging effects on immunity will be helpful for post-transplantation care and adjustment of immunosuppressants for aged recipients. A mouse model, using C3H mice as donors and aged/young C57BL/10J mice as recipients, was employed to study aging effects on immunity. The results showed that frequency of myeloid-derived suppressor cells (MDSC) and level of TGF-β was higher in aged mice than in young mice (4.4 ± 1.4% versus 1.6 ± 1.1%, *p* = 0.026 for MDSC; 21.04 ± 3.91 ng/ml versus 15.26 ± 5.01 ng/ml, *p* = 0.026 for TGF-β). *In vivo*, skin allograft survived longer on the aged than on young mice (19.7 ± 5.2 days versus 11.9 ± 4.1 days, *p* = 0.005). When entinostat was applied to block MDSC, the survival of skin allografts on aged mice was shorten to 13.5 ± 4.7 days which was not different from the survival on young mice (p = 0.359). In conclusion, allogeneic immunity was different in aged from young mice in high frequency of MDSC and high serum level of TGF-β. Blocking the function of MDSC reversed the low immunity in aged mice and caused skin allograft rejection similar to young recipients.

## INTRODUCTION

Organ transplantation is widely accepted as the final treatment for the patients with a specific organ failure or even multi-organ failure. In solid organ transplantation, graft/patient survival is much improved recently because of the advances in immunosuppressive management, surgical techniques and peri-operative care. Among these advances, immunosuppressive management is most important because it is not only employed to prevent acute rejection in short-term but also contribute to long-term graft/patient survival. However, heavy loading of immunosuppressants may be complicated with adverse effects including neurotoxicity, nephrotoxicity, hypertension, hyperglycemia, bone marrow suppression, gastrointestinal disturbance, and so on [1–3]. Heavy immunosuppressants may also inhibit hosts’ defensive immunity, increase risk of opportunistic infections and induce development of neoplasms [4–6]. Therefore, suitable levels of immunosuppressive agents are essential to keep allografts with good function and prevent the adverse effects of organ transplantation [7].

Currently, the aged people are increasing. Many aged people have organ transplantation. In OPTN/SRTR report in 2015, liver transplant recipients above aged 65 years were increased from 9.2% in 2003 to 16.3% in 2013 [8]. The immunity in aged people is weaker than that in young people [9]. In the literature, a narrower repertoire of T-cells and senescent T-cells to decrease the defending abilities to viral infection were reported [10, 11]. Therefore, the dosage of immunosuppressive agents should be minimized in aged people to avoid over-immunosuppression-related morbidities [12]. However, what are the basic reasons beyond T-cells that immunosuppressive agents can be minimized for the aged people is not clearly defined.

In this study, we designed a mouse model using aged and young C57BL/10J (B10) mice as recipients to define the aging effects on immunity. The investigation particularly focused on regulatory T-cells and myeloid-derived suppressor cell (MDSC) which were immunomodulatory cells and associated with immunosuppression. We also measured the cytokines contributed to T-helper 1, T-helper 2 and regulatory T-cells development such as IL-4, IL-10, IL-12, IFN-γ, TGF-β, etc. The understanding of aging effects on immunity is helpful for post-transplant care of the aged recipients in the future.

## RESULTS

### Immune cell population in aged mice

**Figure 1:**
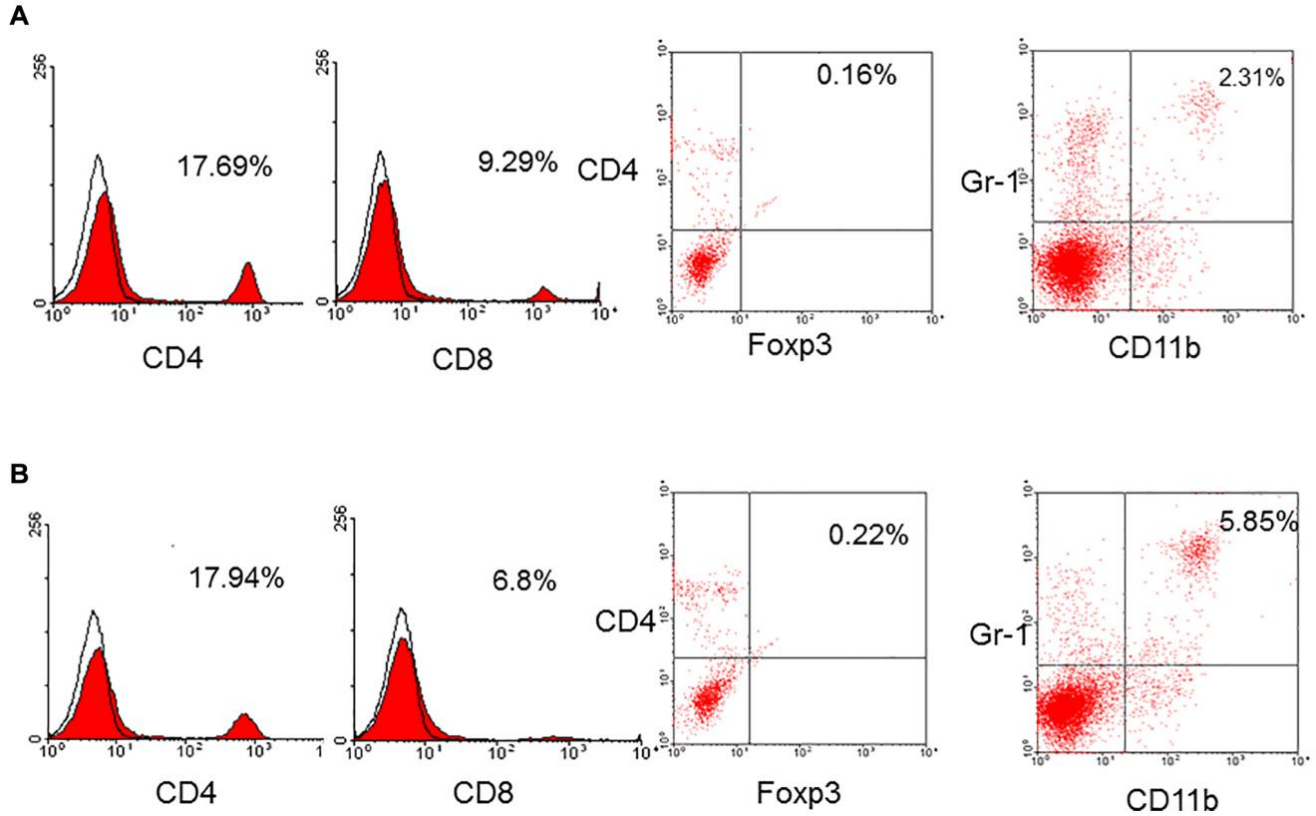
A representative of CD4^+^, CD8^+^, regulatory T-cells (CD4^+^foxp3^+^), and MDSC (CD11b^+^Gr-1^+^) in young (**A**) and aged (**B**) mice. The frequency of CD4^+^ (16.5 ± 1.2% versus 14.6 ± 2.9%, *p* = 0.185), CD8^+^ (8.9 ± 0.7% versus 8.4 ± 1.6%, *p* = 0.324), native regulatory T-cells (1.0 ± 0.8% versus 0.4 ± 0.6%, *p* = 0.160) in aged and young mice was not different. However, the frequency of MDSC (CD11b^+^Gr-1^+^) in aged mice was higher than that in young mice (4.4 ± 1.4% versus 1.6 ± 1.1%, *p* = 0.026).

To determine the alteration of immune cell population in aged mice, the spleens were obtained from aged and young mice to yield spleen cells. The spleen cells were stained by a panel of monoclonal antibodies and analyzed by flow cytometry. The results showed that the frequency of CD4^+^ T-cells in aged mice (*n* = 7) was 14.6 ± 2.9% compared to 16.5 ± 1.2% in young mice (*p* = 0.185) and CD8^+^ T-cells in aged mice was 8.4 ± 1.6% compared to 8.9 ± 0.7% in young mice (*p* = 0.324). The frequency of T-cells was not different between aged and young mice. The frequency of native regulatory T-cells (CD4^+^foxp3^+^) in aged and young mice was not different, either (0.4 ± 0.6% versus 1.0 ± 0.8%, *p* = 0.160). However, the frequency of MDSC (CD11b^+^Gr-1^+^) in aged mice was 4.4 ± 1.4% which was higher than 1.6 ± 1.1% in young mice (*p* = 0.026, Figure 1).

### Cytokine measurements in serum

To determine whether the common cytokines in serum were different in aged and young mice, IL-4, IL-12, IL-10, IFN-γ and TGF-β were measured. The result showed that the level of TGF-β was higher in aged mice than young mice (21.04 ± 3.91 ng/ml versus 15.26 ± 5.01 ng/ml, *p* = 0.026). The levels of IL-4, IL-12, IL-10 and IFN-γ were not different between aged and young mice.

### Immunological reactions of direct antigen presentation in aged recipients

**Figure 2:**
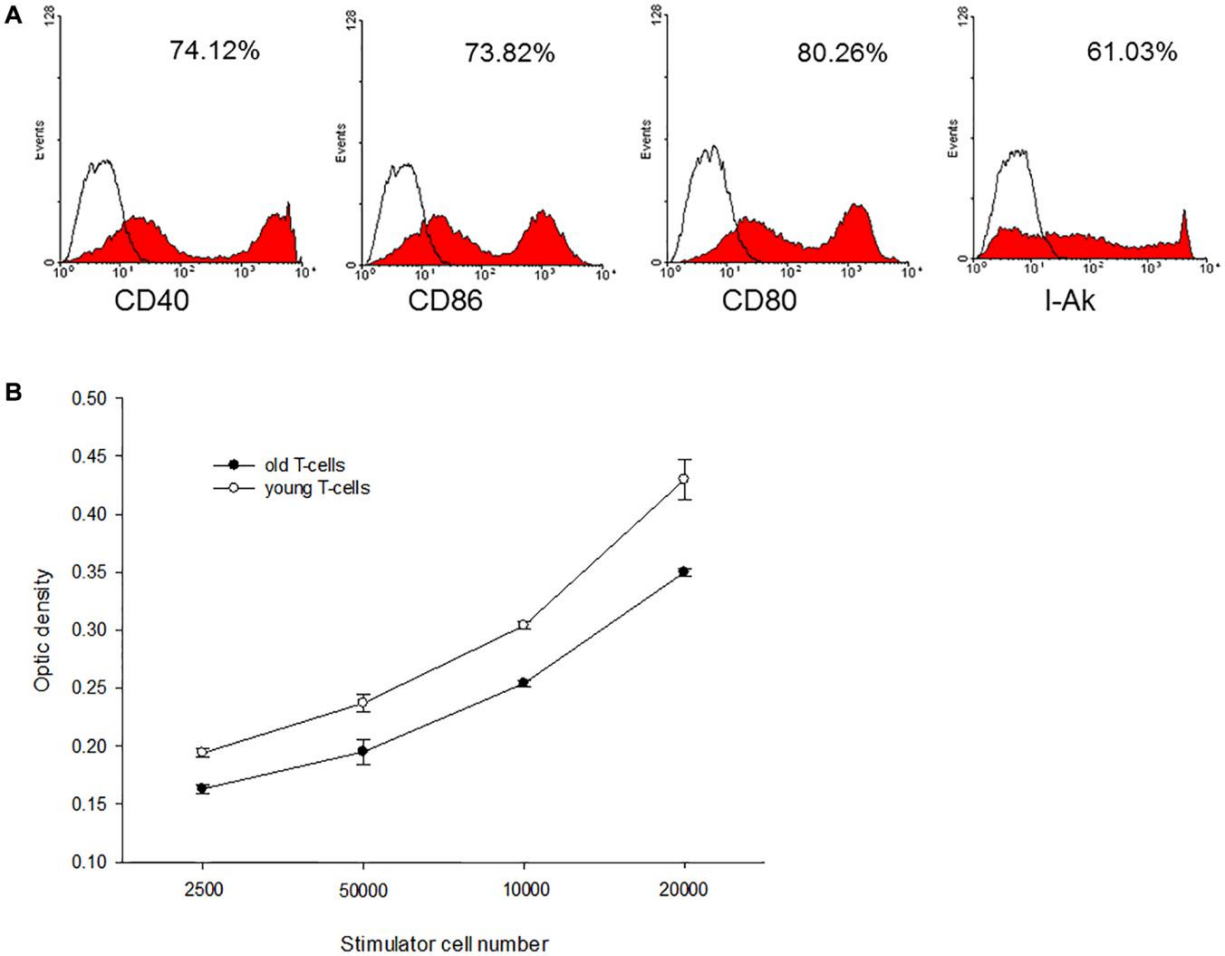
**A representative of C3H mature DC and mixed lymphocyte reactions**. (**A**) The C3H DC cultured in GM-CSF and IL-4 expressed mature phenotype with high levels of CD40, CD80, CD86 and I-A^k^. (**B**) When this mature C3H DC was applied to activate enriched T-cells derived from aged or young B10 mice, MLR showed that proliferation capacity of T-cells from aged mice was lower than that of T-cells from young mice (0.350 ± 0.003 O.D. versus 0.430 ± 0.017 O.D. at responders/stimulatory cells = 100/1, *p* = 0.001).

Bone marrow-derived C3H dendritic cells (DC) were employed as donor antigen-presenting cells and enriched T-cells from aged and young B10 mice were employed as recipients’ cells to perform mixed lymphocyte reaction (MLR). The C3H DC cultured in GM-CSF and IL-4 expressed mature phenotype with high levels of CD40, CD80. CD86 and I-A^k^ (Figure 2A). When this mature C3H DC was applied to activate enriched T-cells derived from aged or young B10 mice, MLR showed that proliferation capacity of T-cells from aged mice was lower than from young mice (0.350 ± 0.003 optic density (O.D.) versus 0.430 ± 0.017 O.D. at responders/stimulatory cells = 100/1, *p* = 0.001) (Figure 2B).

### Cytotoxic abilities of T-cells derived from aged mice

**Figure 3:**
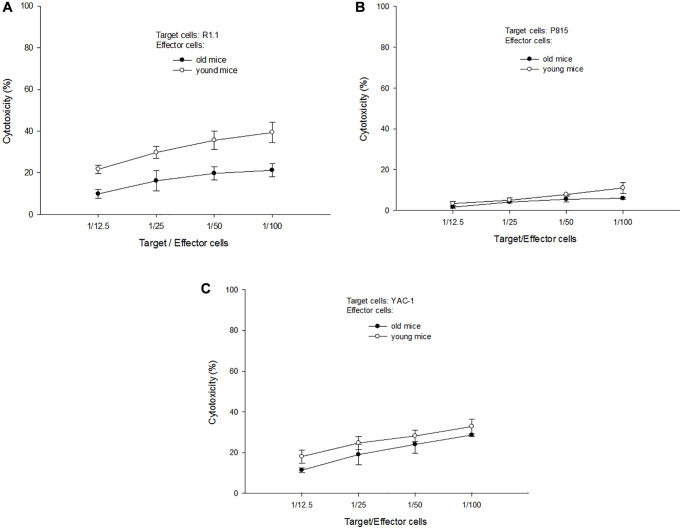
**A representative of cytotoxic tests**. (**A**) When R1.1 cells (H-2^k^, allogeneic) were used as target cells, T-cells derived from aged mice had a lower Ag-specific cytotoxic ability than T-cells derived from young mice (21.2 ± 3.0% versus 39.3 ± 4.8% at target cell/effector cells = 1/100, *p* = 0.003). (**B**) When p815 cells (H-2^d^, third party) were used as target cells, T-cells derived from aged mice had a lower cytotoxic ability than T-cells derived from young mice (6.0 ± 0.6% versus 11.0 ± 2.7% at target cell/effector cells = 1/100, *p* = 0.017). (**C**) When Yac-1 cells (non-Ag-specific, nature killer-sensitive) were used as target cells, the cytotoxic ability of T-cells derived from aged or young mice were not different (28.6 ± 0.6% versus 32.8 ± 3.5% at target cell/effector cells = 1/100, *p* = 0.100).

T-cells derived from aged and young B10 mice were activated by C3H DC for 3 days and employed as effector cells to perform antigen (Ag)-specific and non-specific cytotoxic abilities. R1.1 cells (H-2^k^, allogeneic), p815 cells (H-2^d^, third party) and Yac-1 cells (non-Ag-specific, nature killer-sensitive) were used as target cells. The results showed that T-cells derived from aged mice had a lower Ag-specific cytotoxic ability than T-cells derived from young mice (21.2 ± 3.0% versus 39.3 ± 4.8% at target cell/effector cells = 1/100, *p* = 0.003) (Figure 3A). Even for the third party targets, T-cells derived from aged mice also had a lower cytotoxic ability than T-cells derived from young mice (6.0 ± 0.6% versus 11.0 ± 2.7% at target cell/effector cells = 1/100, *p* = 0.017, Figure 3B). For non-specific targets, the cytotoxic ability of T-cells derived from aged or young mice were not different. (28.6 ± 0.6% versus 32.8 ± 3.5% at target cell/effector cells = 1/100, *p* = 0.100, Figure 3C).

### Inducible regulatory T-cells in aged mice

**Figure 4:**
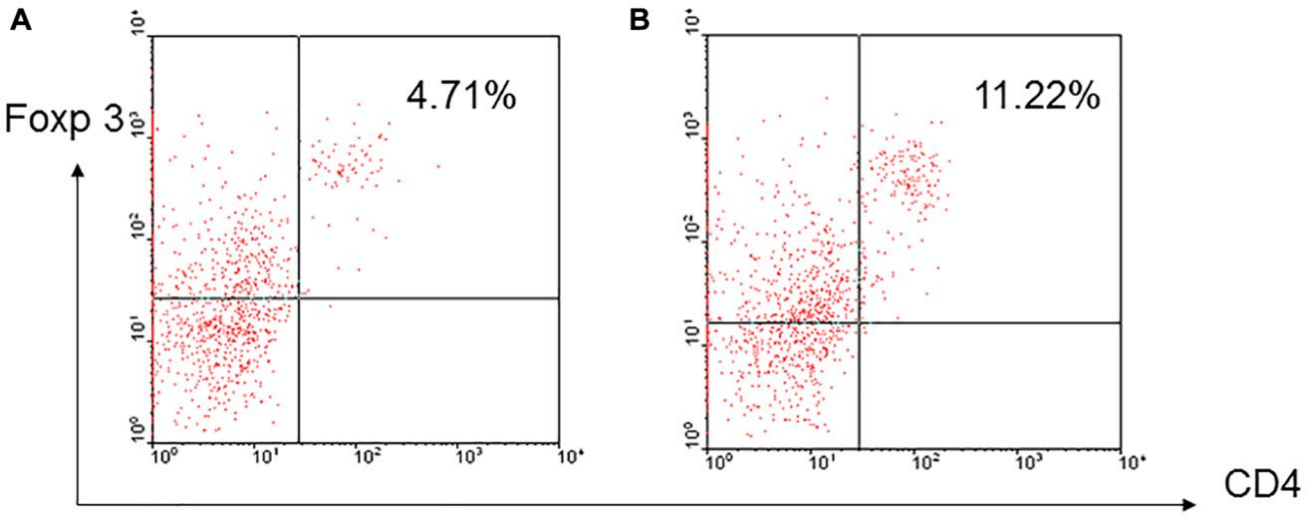
**A representative of induced regulatory T-cells.** Enriched T-cells derived from aged and young B10 mice were activated by C3H DC for 3 days, a higher frequency of CD4^+^foxp3^+^ regulatory cells was induced in the T-cells derived from aged mice (**B**) than young mice (**A**) (7.87 ± 3.42% versus 5.04 ± 2.71%, *p* = 0.023).

To determine whether regulatory T-cells could be induced *in vitro*, enriched T-cells derived from aged and young B10 mice were activated by C3H DC for 3 days and CD4^+^foxp3^+^ cells were examined. The results showed that higher frequency of CD4^+^foxp3^+^ regulatory cells was induced in T-cells derived from aged mice than young mice (7.87 ± 3.42% versus 5.04 ± 2.71%, *p* = 0.023) (Figure 4).

### Immunological reactions of indirect antigen presentation in aged recipients

**Figure 5:**
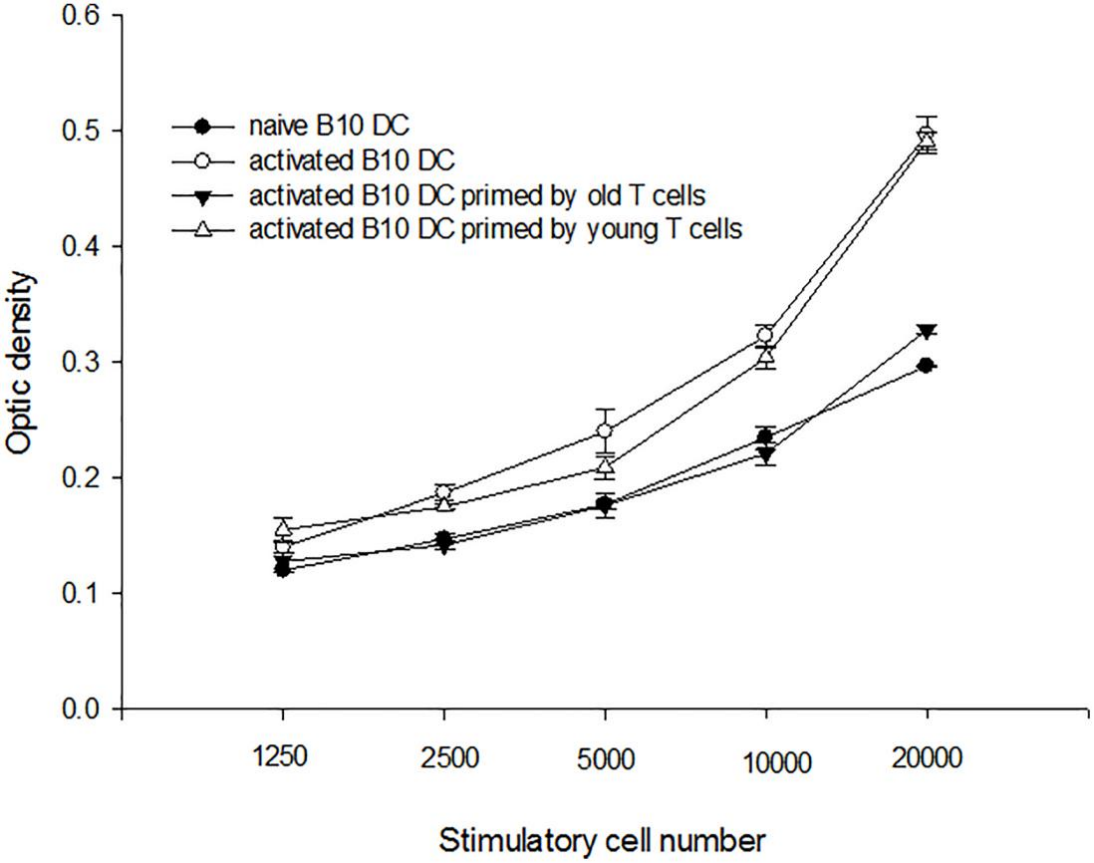
**A representative mixed lymphocyte reaction of indirect antigen presentation**. B10 DC was pulsed with C3H splenocyte lysate overnight and used as stimulators to activate B10 enriched T-cells. The result showed that the proliferating capacities of T-cells from aged were lower than those of T-cells from young mice (0.328 ± 0.004 O.D. versus 0.491 ± 0.008 O.D. at responders/stimulatory cells = 100/1, *p* = 0.001).

To determine whether immune reactions with indirect antigen presentation was altered in aged mice, C3H DC and B10 aged/young enriched T-cells were put in a transwell of B10 DC cultural system. B10 DC was pulsed with C3H splenocyte lysate overnight and used as stimulators to activate B10 enriched T-cells. The result showed that the proliferating capacities of T-cells activated by B10 DC with C3H DC and aged B10 T-cells in transwell were lower than B10 DC with C3H DC and young B10 T-cells in transwell (0.328 ± 0.004 O.D. versus 0.491 ± 0.008 O.D. at responders/stimulatory cells = 100/1, *p* = 0.001) (Figure 5).

### Survival of skin allograft survival

**Figure 6:**
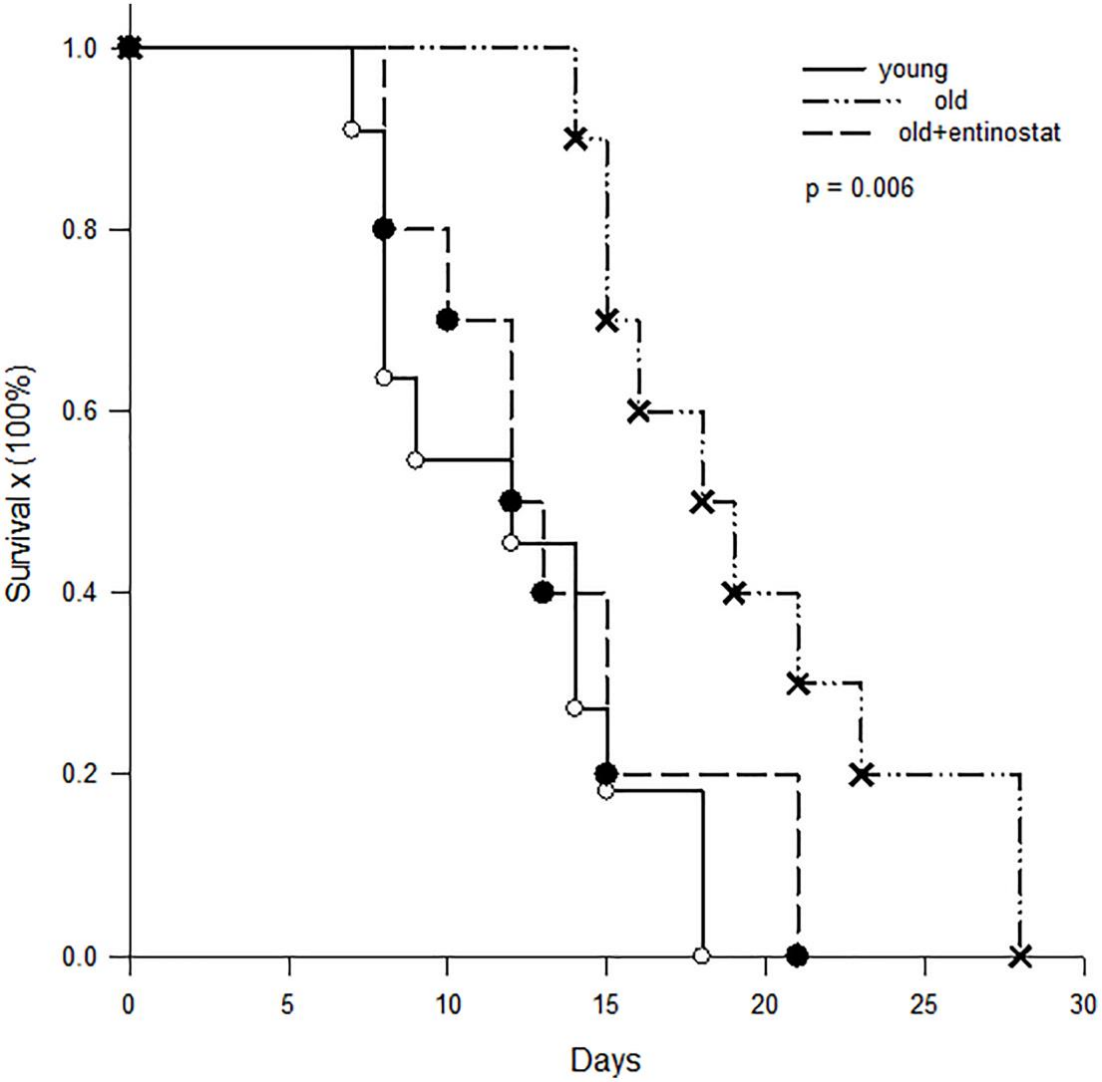
**Kaplan-Meier survival curve of skin allografts**. Skin grafts from C3H mice were transplanted to young (*n* = 11) B10 mice, aged B10 mice (*n* = 10) and aged B10 mice (*n* = 10) fed with entinostat. The results showed that the skin allograft survival on young B10 mice was 11.9 ± 5.2 days and was prolonged to 19.7 ± 5.2 days on aged mice (*p* = 0.005). When the aged mice was fed with entinostat to inhibit MDSC, the survival of skin grafts was shorten to 13.5 ± 4.7 days, which was not different from the survival on young mice (*p* = 0.359).

To determine whether allograft survival was different in aged and young recipients, skin grafts from C3H mice were transplanted to aged (*n* = 20) and young (*n* = 11) B10 mice and survival time was recorded. To determine whether MDSC played an important role of immunosuppression in aged mice, a half of aged mice were fed with entinostat (50 mg/kg/week, Selleck Chemicals, Houston, Tx) after skin transplantation. The results showed that the skin allograft survival on young B10 mice was 11.9 ± 5.2 days and was prolonged to 19.7 ± 5.2 days on aged mice (*p* = 0.005). When the aged mice was fed with entinostat to inhibit MDSC, the survival of skin grafts was shorten to 13.5 ± 4.7 days, which was significantly shorter than the survival on the aged mice without entinostat feeding (*p* = 0.042) and not different from the survival on young mice (*p* = 0.359, Figure 6).

## DISCUSSION

The different frequency of immune cells in aged and young mice was mainly in MDSC. By analyzing the immune cells in the spleen, the frequency of CD4^+^ T-cell, CD8^+^ T-cells and regulatory T-cells was not different between aged and young mice. But, the frequency of MDSC in aged mice was higher than in young mice. MDSC is a well-known immune-suppressor cell which is associated with cancer, sepsis, chronic inflammation, trauma, etc [13, 14]. While the frequency of MDSC increases, the immunity of the hosts is suppressed. Therefore, this study showed that the skin allografts were rejected more slowly in aged mice than young mice. While the function of MDSC was neutralized by entinostat (a class I-specific histone deacetylase inhibitor) [15], the rejection of allogeneic skin grafts in aged mice was accelerated and was similar to the survival in young mice. This reflected the immunologic suppression in aged mice by increasing frequency of MDSC.

The proliferating capacities of T-cells were decreased in aged mice. According to the results in this study, the proliferation of T-cells from aged mice was lower than those from young mice when the T-cells were stimulated by allogeneic dendritic cells. In Shen’s study, they adoptively transferred young naïve T-cells to aged and young mice and found that the expansion of T-cells was reduced in the microenvironment of aged mice, and T-cell response to allotransplantation *in vivo* was also impaired [9]. They concluded that T-cell immunity was impaired by intrinsic and extrinsic factors. Yager et al. reported that the repertoire diversity in T-cells was declined in aged mice, which impaired CD8+ T-cell response to known immune-dominant epitopes [11]. Thus, proliferating ability of T-cells of aged mice was reduced and the reduction was related to T-cells themselves and environmental factors.

Cytokine profile is one of the environmental factors in aged and young mice. Thus, a panel of cytokines in the serum were measured for aged and young mice. The results showed that the levels of TGF-β in the serum of aged mice were significantly higher than that in young mice. TGF-β is a multi-functional cytokine with the property of immunosuppression. In the microenvironment with a high level of TGF- β dendritic cells expressed low levels of co-stimulatory molecules and decreased the stimulatory capacity on T-cells [16, 17]. Naïve T-cells exposed in a high level of TGF-β would be induced to become regulatory T-cells [18, 19]. In this study, we also found that T-cell proliferation capacity were lower in indirect antigen-presenting pathway when they were stimulated by dendritic cells coming from aged mice compared to young mice. All these results implied that immune cells were suppressed in aged mice which had high serum levels of TGF-β.

Although the phenotypic population of CD4^+^ and CD8^+^ T-cells were not altered in aged mice, the cytotoxic abilities of T-cell coming from aged mice were lower than coming from young mice. *In vitro* study, the results clearly showed that antigen-specific cytotoxicity of aged T-cells was significantly lower than young T-cells. *In vivo* study, skin allografts survived longer in aged recipients than in young recipients. In a model of mice implanted with tumors, Grizzle et al. demonstrated that T-cell cytotoxicity to tumor cells was decreased in aged mice and the tumor implanted in aged mice grew more rapidly than in young mice [20]. They also found that the decline of T-cell cytotoxicity was correlated to accumulation of myeloid-derived suppressor cells. Clearly, T-cell immunity declined along with aging and leaded to prolong skin graft survival in aged mice.

Regulatory T-cells could be induced in aged mice although native regulatory T-cells were not different between aged and young mice. Regulatory T-cells are important cells to modulate immune reactions in the hosts [21]. In organ transplantation, regulatory T-cells are believed to induce infectious tolerance [22, 23]. Currently, *ex vivo* expansion of regulatory T-cells has been applied to induce tolerance in clinical trials [24]. In this study, regulatory T-cells were easier to be induced from T-cells derived from aged mice than from young mice. The true mechanism was not known. However, the higher serum level of TGF-β in aged mice may prime the property of regulatory T-cells and contributed to following induction. It was reported that TGF-β played a critical role of regulatory T-cell development in thymus, but also could induce regulatory T-cells in peripheral [18]. The higher level of TGF-β implied that T-cell-mediated immunity could be suppressed easier in aged mice than in young mice and the grafts survival was prolonged.

In conclusion, allogeneic immunity was lower in aged than in young mice evidenced by a higher frequency of MDSC, higher serum level of TGF-β, decreased function of T-cells, and easy-to-induced regulatory T-cells in aged mice. Blocking the function of MDSC by entinostat reversed the low immunity in aged mice and caused skin allograft rejection similar to young recipients. Taking together, the cellular immunity is weaker in aged mice than in young mice and MDSC plays the important role. Based on these experimental results, the clinical regimen of immunosuppression induction after transplantation for aged recipients should be adjusted to prevent or decrease adverse effects.

## MATERIALS AND METHODS

### Mice

Male C3H (H-2^k^, I-A^k^) and C57BL/10J (B10; H-2^b^, I-A^b^) mice, 8 to 10-week-old were purchased from the Animal Laboratory of National Institute Taipei, Taiwan, and maintained in the pathogen-free facility at Change-Gung Memorial Hospital. Some B10 mice were maintained to 12 months old to be employed as the aged mice. Experimental use of these mice was according to the guidelines for ethics and experiments on animals and was approved by Animal Care Committee of Chang-Gung Memorial Hospital (No. 105-0293C).

### Bone marrow-derived dendritic cells propagation

To propagate bone marrow-derived dendritic cells (DC), bone marrow cells were harvested from femurs and tibias of C3H or B10 mice, and cultured in 24-well plates (2 × 10^6^ cells/well) in 1 ml of RPMI-1640 medium (Life Technologies, Graitherburg, MD, USA), supplemented with 4 ng/ml recombinant mouse GM-CSF (R&D Systems Inc. Minneapolis, MN, USA) and 1000 u/ml mouse IL-4 (R&D Systems Inc. Minneapolis, MN, USA). The propagation of large numbers of DC from mouse bone marrow with minor modification herein was similar to that described initially by Inaba et al. [25]. The DC progenitor clusters were selected after 2 days of culture by gently swirling the plates and depleting the non-adherent granulocytes. Half of the cultural medium was refreshed. The DC was harvested after 5 days of culture.

### Enriched T-cells

Enriched T-cells were obtained from splenocytes passing through nylon wool column. Nylon wool column was prepared by packing 0.5 gram nylon wool in a 10-c.c. syringe. The column was equilibrated by running cultural medium and incubated at 37°C for an hour before splenocytes were loaded into nylon wool column. Splenocyte-loaded nylon wool column was incubated at 37°C for one hour. Then, the non-adherent cells, enriched T-cells, were collected.

### Cell lines

Murine cancer cell lines were employed in *in vitro* studies. R1.1, P815, Yac-1 cell lines were obtained from the Cell Collection and Research Center (CCRC, Hsin-Chu, Taiwan), maintained in antibiotic-free Dulbecco‘s minimal essential Medium (DMEM, Life Technologies, Gaithersburg, MD, USA) and supplemented with 10% v/v fetal bovine serum.

### Phenotypes of immune cells

The phenotypes of the cells were classified by the expression of surface molecules. After the surface molecules were stained directly by a panel of fluorescence monoclonal antibodies, the cell surface molecular expressions of DC, myeloid-derived suppressor cells (MDSC) and regulatory T-cells (Treg) were analyzed by cytofluorography employing a Beckman Coulter NAVIOS flow cytometer (Beckman Coulter Co., Indianapolis, IN, USA). The fluorescence monoclonal antibodies included phycoerythrin (PE)-conjugated anti-CD40, CD80, CD86 and I-A^k^ (PharMingen, San Diego, CA, USA) for DC, phycoerythrin (PE)-conjugated anti-Gr-1 monoclonal antibodies and fitc-conjugated anti-CD11b monoclonal antibody (PharMingen, San Diego, CA, USA) for MDSC, and anti-foxp3 for regulatory T-cells (eBioscience, Thermo Fisher Scientific Inc., USA).

### Quantitative T-cell proliferation

The allogeneic stimulatory capacities of DC were determined via an one-way mixed lymphocyte reaction (MLR) employing colorimetric tetrazolium (MTT) assay [26, 27]. Enriched T-cells were stimulated by irradiated DC triplicatedly in 96-well plates for 3 days. In the last 4 hours of the procedure, sterilized MTT (3-(4,5-dimethylthiazol-2-yl)-2,5-diphenyl tetrazolium bromide) (5 mg/ml, Sigma, St Louis, MO, USA) was added to each well (10 ul/well) to produce blue formazan. Upon termination of MLR, acid-isopropanol (0.04N HCL-isopropanol, 100 ul/well) was added to each well and mixed thoroughly to dissolve the blue crystals. After 10 minutes at room temperature to ensure that all crystals were dissolved, the plates were read on a Dynatech MR580, microelisa reader, employing a test wavelength of 570 nm to measure the optical densities (OD) of formazan formation.

### Cell-mediated cytotoxicity

T-cells activated by DC for three days were applied as effector cells. P815 (H-2^d^), R1.1 (H-2^k^), and YAC-1 (natural killer sensitivity) cells were applied as targets. Cell-mediated cytotoxicity was performed at various effector-to-target ratio triplicatedly in 96-well plates, and assessed by flow cytometry. To assess the cell-mediated cytotoxicity by flow cytometry, the targets were labeled with PKH-26 and 5-(and-6)-carboxyfluorescein diacetate succinimidyl ester (CFSE) according to the manufacturer’s instruction (Sigma. St. Louis, MO, USA). Briefly, target cells, 1 × 10^6^/ml, were stained with PKH-26 (final concentration of 2.5 × 10^-6^ M) at room temperature for 5 minutes and washed by PBS once. Then, these PKH-26 labeled target cells were stained with CFSE (final concentration of 5 × 10^-6^ M). After 4 hours of cytotoxic reaction, all cells were collected and analyzed by flow cytometry. The target cells were identified by PKH-26 positive gate. The cells contained within the PKH-26 and CFSE^high^ double positive gate represent viable target cells. Therefore, percent survival of target cells = (CFSE^high^ percent of test well/CFSE^high^ percent of spontaneous release) × 100%. Percent specific cytotoxicity = (1-% survival) ×100%.

### Cytokine measurement

The serum of young and aged mice was collected. The product of cytokines was measured by enzyme-linked immunoabsorbent assay. The procedure was conducted as the instructions of producers. (PharMingen, San Diego, CA, USA).

### Skin transplantation

Under adequate anesthesia, an incision was made on the flank of B10 mice. A 1 × 1 cm skin flap taken from C3H mice was attached to the flank of B10 mice and fixed to adjacent skin with 3–0 Dexon sutures. The skin flap was protected by circulated gauge for 3 days. The skin flap was observed every 2–3 days. Rejection was diagnosed when the skin grafts were fully detached.

### Statistical analysis

Unpaired Student’s *t*-test was used to analyze continuous variables. Categorical variables were analyzed by either Chi-square test or Fisher’s exact test. All pairwise multiple comparisons were done by Holm-Sidak method. The survival rates were calculated using the Kaplan-Meier method. The statistical analyses were all performed with SigmaPlot 12.3 for Window software (Systat Software, Inc., San Jose, CA, USA). *P* < 0.05 was considered statistically significant.

## References

[1] Pillai AA, Levitsky J. Overview of immunosuppression in liver transplantation. World J Gastroenterol. 2009; 15:4225–33. 10.3748/wjg.15.4225 19750565 PMC2744178

[2] Dunn CJ, Wagstaff AJ, Perry CM, Plosker GL, Goa KL. Cyclosporin: an updated review of the pharmacokinetic properties, clinical efficacy and tolerability of a microemulsion-based formulation (neoral)1 in organ transplantation. Drugs. 2001; 61:1957–2016. 10.2165/00003495-200161130-00006 11708766

[3] Scott LJ, McKeage K, Keam SJ, Plosker GL. Tacrolimus: a further update of its use in the management of organ transplantation. Drugs. 2003; 63:1247–97. 10.2165/00003495-200363120-00006 12790696

[4] Rodríguez-Perálvarez M, De la Mata M, Burroughs AK. Liver transplantation: immunosuppression and oncology. Curr Opin Organ Transplant. 2014; 19:253–60. 10.1097/MOT.0000000000000069 24685671 PMC4025587

[5] Myint TM, Turner RM, Craig JC, Cross NB, Kable K, Nankivell BJ, Chapman JR, Webster AC, O'Connell P, Dwyer DE, Jeoffreys N, Roger SD, Wong G. Test performance characteristics of quantitative nucleic acid testing for polyomaviruses in kidney and kidney-pancreas transplant recipients. Clin Transplant. 2013; 27:E571–79. 10.1111/ctr.12195 23952788

[6] Dantal J, Campone M. Daunting but Worthy Goal: Reducing the De Novo Cancer Incidence After Transplantation. Transplantation. 2016; 100:2569–83. 10.1097/TP.0000000000001428 27861286

[7] Farkas SA, Schnitzbauer AA, Kirchner G, Obed A, Banas B, Schlitt HJ. Calcineurin inhibitor minimization protocols in liver transplantation. Transpl Int. 2009; 22:49–60. 10.1111/j.1432-2277.2008.00796.x 19121146

[8] Kim WR, Lake JR, Smith JM, Skeans MA, Schladt DP, Edwards EB, Harper AM, Wainright JL, Snyder JJ, Israni AK, Kasiske BL. OPTN/SRTR 2015 Annual Data Report: Liver. Am J Transplant. 2017 (Suppl 1); 17:174–251. 10.1111/ajt.14126 28052604

[9] Shen H, Tesar BM, Du W, Goldstein DR. Aging impairs recipient T cell intrinsic and extrinsic factors in response to transplantation. PLoS One. 2009; 4:e4097. 10.1371/journal.pone.0004097 19119314 PMC2606020

[10] Abecassis M, Bridges ND, Clancy CJ, Dew MA, Eldadah B, Englesbe MJ, Flessner MF, Frank JC, Friedewald J, Gill J, Gries C, Halter JB, Hartmann EL, et al. Solid-organ transplantation in older adults: current status and future research. Am J Transplant. 2012; 12:2608–22. 10.1111/j.1600-6143.2012.04245.x 22958872 PMC3459231

[11] Yager EJ, Ahmed M, Lanzer K, Randall TD, Woodland DL, Blackman MA. Age-associated decline in T cell repertoire diversity leads to holes in the repertoire and impaired immunity to influenza virus. J Exp Med. 2008; 205:711–23. 10.1084/jem.20071140 18332179 PMC2275391

[12] Heldal K, Midtvedt K. Managing transplant rejection in the elderly: the benefits of less aggressive immunosuppressive regimens. Drugs Aging. 2013; 30:459–66. 10.1007/s40266-013-0082-z 23609876

[13] Gabrilovich DI, Nagaraj S. Myeloid-derived suppressor cells as regulators of the immune system. Nat Rev Immunol. 2009; 9:162–74. 10.1038/nri2506 19197294 PMC2828349

[14] Youn JI, Gabrilovich DI. The biology of myeloid-derived suppressor cells: the blessing and the curse of morphological and functional heterogeneity. Eur J Immunol. 2010; 40:2969–75. 10.1002/eji.201040895 21061430 PMC3277452

[15] Orillion A, Hashimoto A, Damayanti N, Shen L, Adelaiye-Ogala R, Arisa S, Chintala S, Ordentlich P, Kao C, Elzey B, Gabrilovich D, Pili R. Entinostat Neutralizes Myeloid-Derived Suppressor Cells and Enhances the Antitumor Effect of PD-1 Inhibition in Murine Models of Lung and Renal Cell Carcinoma. Clin Cancer Res. 2017; 23:5187–201. 10.1158/1078-0432.CCR-17-0741 28698201 PMC5723438

[16] Tu E, Chia PZ, Chen W. TGFβ in T cell biology and tumor immunity: Angel or devil? Cytokine Growth Factor Rev. 2014; 25:423–35. 10.1016/j.cytogfr.2014.07.014 25156420 PMC4182110

[17] Esebanmen GE, Langridge WHR. The role of TGF-beta signaling in dendritic cell tolerance. Immunol Res. 2017; 65:987–94. 10.1007/s12026-017-8944-9 28845509

[18] Hadaschik EN, Enk AH. TGF-β1-induced regulatory T cells. Hum Immunol. 2015; 76:561–64. 10.1016/j.humimm.2015.06.015 26116540

[19] Sanjabi S, Oh SA, Li MO. Regulation of the Immune Response by TGF-β: From Conception to Autoimmunity and Infection. Cold Spring Harb Perspect Biol. 2017; 9:a022236. 10.1101/cshperspect.a022236 28108486 PMC5453394

[20] Grizzle WE, Xu X, Zhang S, Stockard CR, Liu C, Yu S, Wang J, Mountz JD, Zhang HG. Age-related increase of tumor susceptibility is associated with myeloid-derived suppressor cell mediated suppression of T cell cytotoxicity in recombinant inbred BXD12 mice. Mech Ageing Dev. 2007; 128:672–80. 10.1016/j.mad.2007.10.003 18036633

[21] Sakaguchi S, Miyara M, Costantino CM, Hafler DA. FOXP3+ regulatory T cells in the human immune system. Nat Rev Immunol. 2010; 10:490–500. 10.1038/nri2785 20559327

[22] Newell KA, Phippard D, Turka LA. Regulatory cells and cell signatures in clinical transplantation tolerance. Curr Opin Immunol. 2011; 23:655–59. 10.1016/j.coi.2011.07.008 21982510

[23] Hippen KL, Riley JL, June CH, Blazar BR. Clinical perspectives for regulatory T cells in transplantation tolerance. Semin Immunol. 2011; 23:462–68. 10.1016/j.smim.2011.07.008 21820917 PMC3230779

[24] Tang Q, Bluestone JA. Regulatory T-cell therapy in transplantation: moving to the clinic. Cold Spring Harb Perspect Med. 2013; 3:a015552. 10.1101/cshperspect.a015552 24186492 PMC3808774

[25] Inaba K, Inaba M, Romani N, Aya H, Deguchi M, Ikehara S, Muramatsu S, Steinman RM. Generation of large numbers of dendritic cells from mouse bone marrow cultures supplemented with granulocyte/macrophage colony-stimulating factor. J Exp Med. 1992; 176:1693–702. 10.1084/jem.176.6.1693 1460426 PMC2119469

[26] Mosmann T. Rapid colorimetric assay for cellular growth and survival: application to proliferation and cytotoxicity assays. J Immunol Methods. 1983; 65:55–63. 10.1016/0022-1759(83)90303-4 6606682

[27] Denizot F, Lang R. Rapid colorimetric assay for cell growth and survival. Modifications to the tetrazolium dye procedure giving improved sensitivity and reliability. J Immunol Methods. 1986; 89:271–77. 10.1016/0022-1759(86)90368-6 3486233

